# Effects of the ketogenic diet in mice with hind limb ischemia

**DOI:** 10.1186/s12986-022-00695-z

**Published:** 2022-08-29

**Authors:** Adilan Shalamu, Zhen Dong, Bowen Liu, Lihong Pan, Yun Cai, Liwei Liu, Xiurui Ma, Kai Hu, Aijun Sun, Junbo Ge

**Affiliations:** 1 Department of Cardiology, Zhongshan Hospital, Shanghai Institute of Cardiovascular Diseases, Fudan University, Shanghai, China; 2 Key Laboratory of Viral Heart Diseases, National Health Commission, Shanghai, China; 3 Key Laboratory of Viral Heart Diseases, Chinese Academy of Medical Sciences, Shanghai, China; 4 Institutes of Biomedical Sciences, Fudan University, Shanghai, China; 5 Department of Cardiology, Shanxi Cardiovascular Hospital, Taiyuan, 030024 China

**Keywords:** Ketogenic diet, Hind limb ischemia, Blood perfusion, Tissue recovery

## Abstract

**Background:**

The ketogenic diet (KD) has anti-tumor and anti-diabetic effects in addition to its anti-epileptic role. It could also improve cardiac function and attenuate neurological insult. However, the effect of KD on blood perfusion or tissue recovery after ischemia remains largely unknown. Thus, we observed blood flow and ischemic tissue recovery following hind limb ischemia (HLI) in mice.

**Methods:**

C57 mice were fed with either a KD or normal diet (ND) for 2 weeks, before inducing hind limb ischemia, blood perfusion of ischemic limb tissue was observed at 0, 7, and 21 days post operation.

**Results:**

KD not only decreased blood perfusion of ischemic limb tissue but also delayed muscle recovery after ischemia, induced muscle atrophy of non-ischemic tissue compared to mice fed with ND. Furthermore, KD delayed wound healing at the surgical site and aggravated inflammation of the ischemic tissue. At the cellular level, KD altered the metabolic status of limb tissue by decreasing glucose and ketone body utilization while increasing fatty acid oxidation. Following ischemia, glycolysis, ketolysis, and fatty acid utilization in limb tissue were all further reduced by KD, while ketogenesis was mildly increased post KD in this mice model.

**Conclusion:**

The KD may cause impaired tissue recovery after ischemia and possible muscle atrophy under a prolonged diet. Our results hint that patients with limb ischemia should avoid ketogenic diet.

**Supplementary Information:**

The online version contains supplementary material available at 10.1186/s12986-022-00695-z.

## Introduction

The ketogenic diet (KD) is a low-carbohydrate diet that is high in fat and strictly limits the intake of sugars, which is recognized as an effective anti-epileptic treatment. In 1911, Gulep and Marie recorded starvation as a treatment for epilepsy [1]. After studying metabolic changes under starvation later in 1921, Woody-Att noted that acetone and beta-hydroxybutyric acid appear in normal subjects through starvation or a diet containing too low a proportion of carbohydrate and too high a proportion of fat; therefore, what was termed a “ketogenic diet” was widely used throughout the 1920s and the 1930s [1, 2]. Along with the discovery of new medicines for epilepsy, KD then became a last treatment option for epilepsy. In addition, KD became widely used as a treatment for obesity in the 1970s.

Ketone bodies, are short-chain fatty acids produced during the β-oxidation of fatty acids in the liver and delivered to extrahepatic tissues for energy supply by blood circulation. When the body encounters starvation or extreme exercise, the liver starts to produce ketone bodies, thereby increasing the uptake and usage of ketones by extrahepatic tissues (especially the brain, heart, and skeletal muscles) for energy supply [3]. Aside from the starvation state, hyperketonemia has also been found in diabetes, pregnancy, neonatal period, and adherence to low carbohydrate diets [4]. Circulating total ketone body concentrations in healthy adult humans are usually within 100 to 250 µM, and rise to ~ 1 mM after prolonged exercise or 24 h of fasting, and can be as high as 20 mM in pathological states such as diabetic ketoacidosis [5, 6].

A new perspective on KD has recently emerged due to observed anti-cancer and anti-diabetic effects. Numerous studies have reported improvement of diabetes by lowering body weight and blood glucose, and by improving insulin resistance. KD has also been reported to provide an insulin secretagogue effect by improving islet cell function [7]. Most studies have reported that KD inhibits the growth of tumors in pancreatic cancer, glioblastoma, and brain cancer [8–10]. Identifying the signaling effects of ketone bodies has introduced a broad trend of further investigation reporting that KD presents a significant neuroprotective and possibly therapeutic effect in non-alcoholic fatty liver disease [11, 12]. In addition, significant improvement of cardiac function in patients with heart failure has drawn attention, suggesting that KD may have therapeutic value in heart failure treatment by increasing blood ketone levels; this finding was further supported by the presence of SGLT2 inhibitors [13–15].

Blood reperfusion by angiogenesis is fundamental to many physiological and pathological processes such as ischemia and inflammation. Myocardial and limb ischemia are common diabetic complications caused by a diseased metabolic environment as hyperglycemia, hyperlipidemia and hyperketonemia. Fasting and calorie restriction also increase blood ketone levels and alter body metabolism similarly to KD by changing the expression of FOXO- and PCG-1-related genes [16]. Calorie-restricted diets reduce angiogenesis, whereas fasting induces angiogenesis by improving endothelial progenitor cells in mice [17–20]. Moreover, lack of ketolysis-related enzymes was found to significantly impair lymph vessel growth in mice [21]. However, how ketone bodies effect blood vessel growth during angiogenesis, and how they affect blood perfusion under ischemic conditions remains unknown. Hind limb ischemia (HLI) is an ideal animal model to investigate vascular regeneration which is also often used to evaluate ischemic perfusion and tissue response. Therefore, we conducted this study to fully observe blood flow and ischemic tissue recovery following hind limb ischemia in mice fed a KD. We found that KD not only reduced blood perfusion in ischemic limb tissue but also induced muscle atrophy and ischemic tissue fibrosis along with delayed wound healing in mice.

## Materials and methods

### Animals and diet

C57BL/6 N male mice aged 8–10 weeks (weight 22–25 g) were purchased from Gem Pharma Tech LLC (Nanjing, Jiangsu, China) and maintained in a 12/12-h light/dark cycle environment at a constant temperature of 22 °C with free access to standard laboratory chow and tap water. They were housed in groups and were divided into normal diet (ND) group and ketogenic diet (KD) group (n = 13 in each group), fed with standard chow and ketogenic chow respectively (Additional file 1: Table S1, purchased from Xietong Pharma Co., Jiangsu, China), with free access to food and water 24hs. After 2 weeks, mice underwent hindlimb ischemia by ligation of the unilateral femoral artery and were given continued access to the two different diets. After the procedure, mice were evaluated for ischemic hindlimb blood perfusion using a laser Doppler perfusion imager. All animal experimental procedures conformed to the Guide for the Care and Use of Laboratory Animals published by the US National Institutes of Health (NIH publication no. 85–23, revised 1996) and were reviewed and approved by the Animal Ethics Committee at Zhongshan Hospital, Fudan University, China.

### Measurement of plasma metabolic parameters

Blood glucose and ketone levels (represented by β-hydroxybutyrate levels) were measured using a glucose meter and ketone meter, respectively (Abbott Diabetes Care, Maidenhead, UK). Tissue β-HB content was measured by β-Hydroxybutyrate (β-HB) assay kits (MAK041, Sigma, Kawasaki, Kanagawa). Tissues were homogenized in cold β-hydroxybutyrate assay buffer and centrifuged at 13,000 g for 10 min at 4 °C to remove insoluble material.

### Hind-limb ischemia procedure

To establish a hind limb ischemia model, we performed unilateral femoral artery ligation in mice. Following percutaneous injection of the anesthetic (4% chloral hydrate), a groin incision was made in the left adductor hind-limb region. The femoral artery was identified, ligated with 6–0 silk ties at the ends of the vascular trunk, and transected from the middle. The incision was then closed with interrupted non-absorbable sutures, and mice were closely monitored for 24 h post-procedure.

### Doppler perfusion

Mice were subjected to an inhaled anesthetic at body temperature maintained by a warming pad. A laser Doppler perfusion imager (Periscan PIM3, Perimed, Beijing, China), was used to evaluate the bilateral hind limbs. Perfusion was evaluated in the whole limb, gastrocnemius region, and hind-paw region on post-HLI days 0, 7, and 21.

### Staining

Gastrocnemius muscle tissues were fixed in 4% paraformaldehyde, dehydrated, and embedded in paraffin then dehydrated in graded ethanol solutions and toluene. Tissues were dissected into 5-μm-thick sections and stained with hematoxylin and eosin (H&E) and Masson. For immunofluorescence staining, the sections were blocked with 10% goat serum albumin (Invitrogen, Waltham, Massachusetts, USA) for 60 min before staining with CD31 monoclonal antibody (1:1500, CST).

### Immunoblotting

Total protein was extracted from the gastrocnemius tissue. Equal amounts of protein extract were separated by SDS-PAGE and transferred to polyvinylidene difluoride membranes. The membranes were blocked with 5% bovine serum albumin and probed with primary antibodies individually at 4 °C overnight. After subsequent washing, the blots were incubated with horseradish peroxidase-coupled anti-rabbit or anti-mouse secondary antibodies at room temperature for 2 h. The blots were visualized and detected by chemiluminescence reaction (LuminataTM Forte, Millipore, Burlington, Massachusetts, USA) and ChemiDoc™ Imaging System (Bio-Rad, Hercules, CA, US). The density of the protein blots was determined using Image J software (1.50i, Open Source, USA) and normalized to β-actin (1:1000, Kang Chen, Wuxi, China).

### RNA procedures

Total RNA from gastrocnemius tissues was extracted using TRIzol™ Reagent (#15,596,026, Invitrogen, Waltham, Massachusetts USA). The concentration and purity of RNA were determined by Nanodrop (Thermo Fischer, Waltham, Massachusetts, USA), and 1000 ng RNA was purified with an A260/A280 ratio of 1.8–2.0 and then reverse transcribed into cDNA using PrimeScript™ Reverse Transcription Master mix (# RR036A, TaKaRa, Kusatsu, Shiga, Japan). Reverse transcription polymerase chain reaction (RT-PCR) was performed using PrimeScript™ RT Master Mix (TaKaRa, Japan). A total of 20 μL reaction system was used, including DNA template 1.6 μL and SYBR 10 μL, and primers 0.4 μL and ddH2O 7.6 μL. Primers used in this study are listed in Additional file 1: Table S2. The PCR reaction cycles were set as follows: 30 s at 95 °C, then 5 s at 95 °C and 30 s at 60 °C for 40 cycles. Fluorescence signals were normalized to Actb using the 2 − ΔΔCT method.

### Statistical analysis

All statistical analyses were performed using Prism 7.0 (GraphPad Software, Inc., La Jolla, CA, USA). Continuous variables are expressed as mean ± standard error of the mean (SEM). Normal distribution was determined using the Shapiro–Wilk test. Differences in normal variates were tested using the Student’s *t*-test (within two groups) or a one-way analysis of variance (ANOVA, among three groups or more), with post hoc comparisons using the Tukey’s multiple comparisons test. Non-normal data were analyzed using the Mann–Whitney U test or the Kruskal–Wallis H test. Statistical significance was defined as two-tailed *P* < 0.05(*), **P < 0.01(**), and ***P < 0.001(***).

## Results

### Ketogenic diet induced weight loss and affected metabolite levels in blood and tissues

**Fig. 1:**
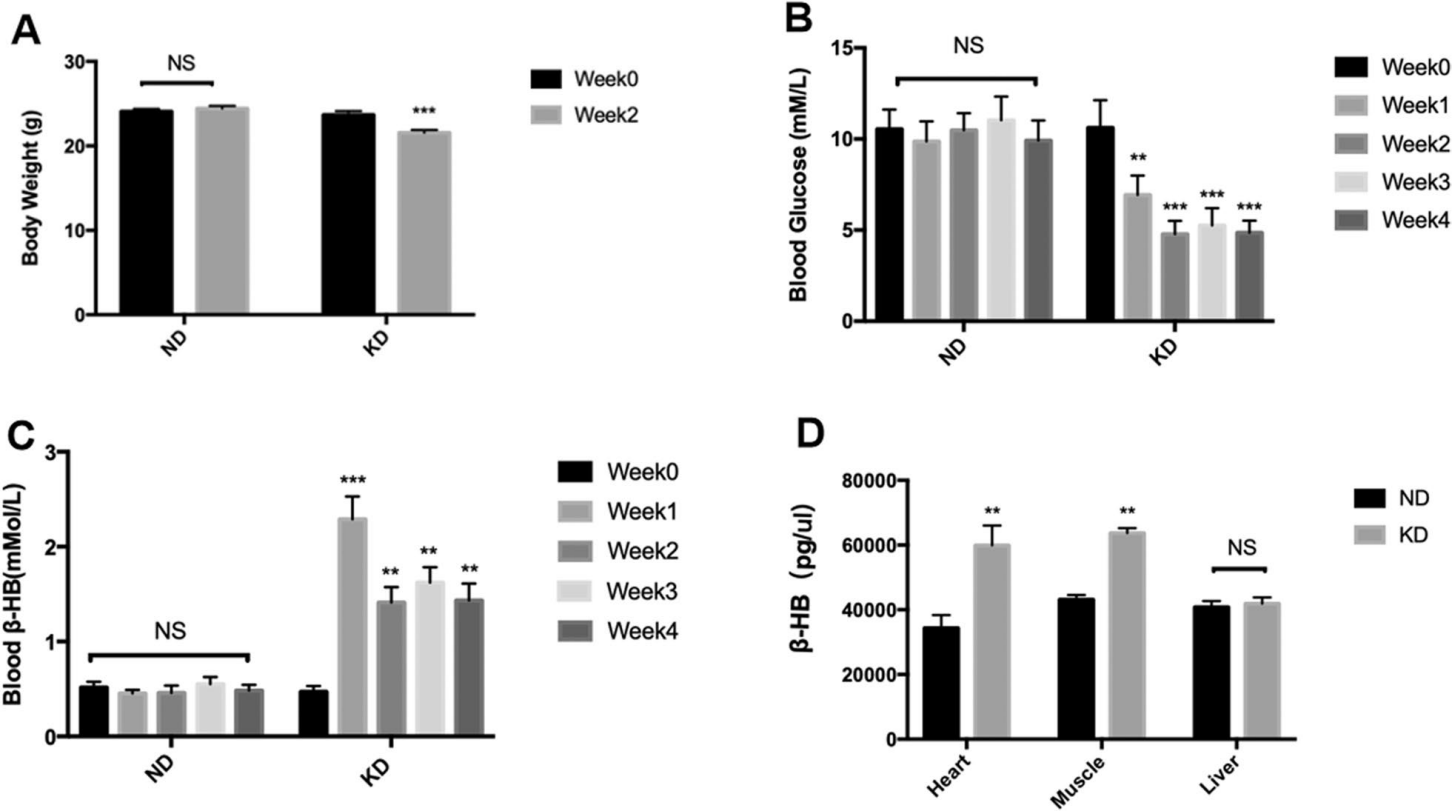
Body weight and blood metabolic parameters: **A** body weight changes of mice in the ND and KD groups during 2 weeks of feeding **B** observation of blood glucose levels during 4 weeks of feeding **C** observation of blood β-hydroxybutyrate level during 4 weeks of feeding **D** β-hydroxybutyrate concentration in heart, muscle and liver tissue. Mean ± SEM, n = 13, *P < 0.05, **P < 0.01, ***P < 0.001

After feeding for 2 weeks, body weight of mice was measured, and was found to be significantly lower in the KD group than in the ND group (Fig. 1A). Random blood glucose and blood ketone levels (represented by blood β-hydroxybutyrate) were also measured using glucose and ketone meters during 4 weeks of feeding. There was no significant change in random blood glucose levels in ND-fed mice over 4 weeks; on the other hand in KD-fed mice, blood glucose gradually decreased in the first 2 weeks then remained stable in the third and fourth weeks (Fig. 1B). Similarly, the blood ketone levels of mice in ND group did not show a significant change when there was a remarkable increase in blood ketone levels of mice in KD group in the first week of feeding, reaching 2.7 mmol/L, then gradually returning to approximately 2 mmol/L in the second week and mostly remaining stable in third and fourth weeks (Fig. 1C). Finally β-hydroxybutyrate content in different tissues was measured including muscle, heart and liver using a β-hydroxybutyrate assay kit, and it was found that β-hydroxybutyrate levels increased in the heart and muscle tissues of mice in the KD group with a greater increase in the heart tissue compared with mice in the ND group. But there was no significant difference in β-hydroxybutyrate content in liver tissues between the two groups (Fig. 1D).

### KD impaired perfusion recovery and revascularization in chronic hind limb ischemia

**Fig. 2:**
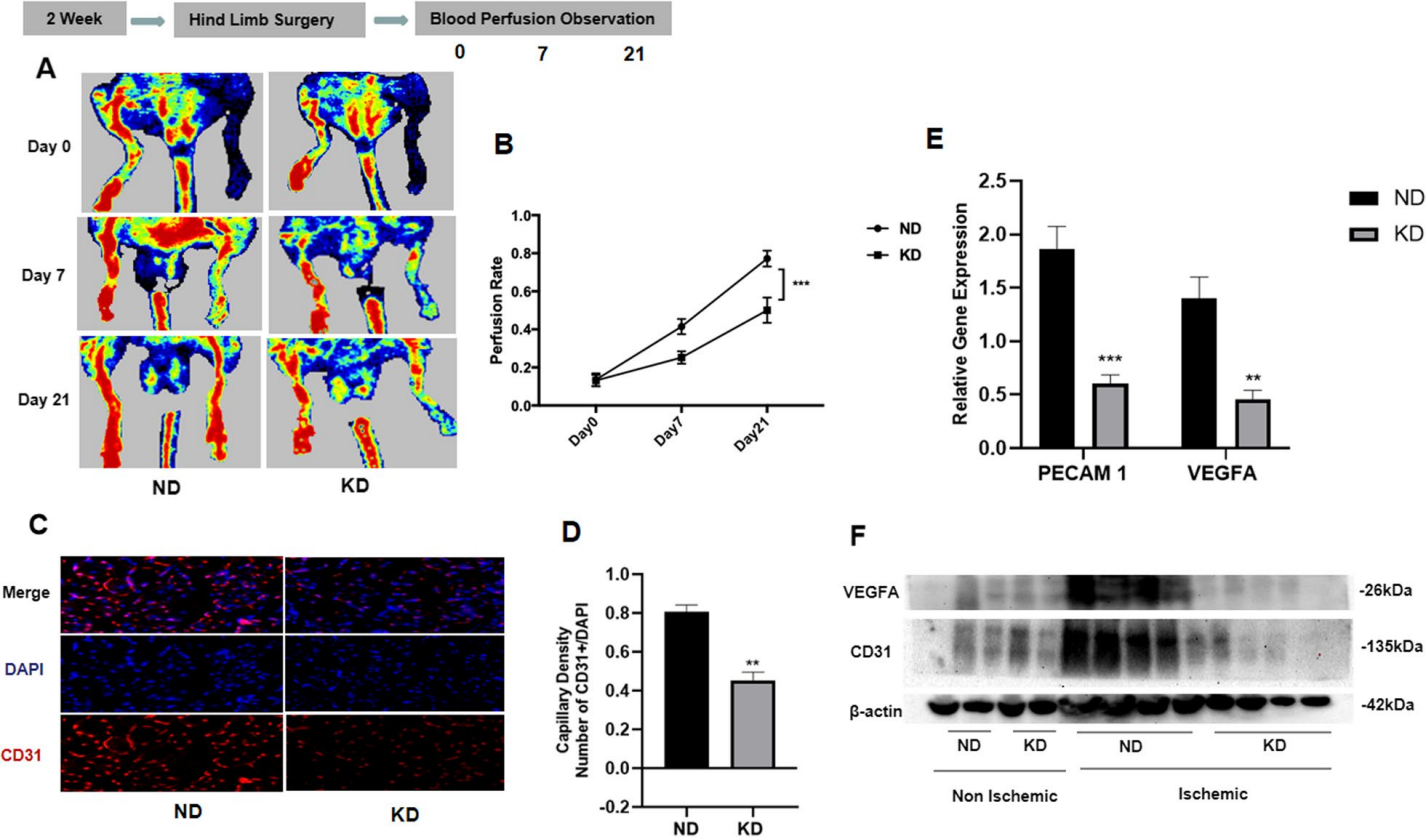
Effects of ketogenic diet on perfusion recovery, revascularization in chronic hindlimb ischemia; **A** original laser Doppler perfusion images displaying hindlimb perfusion 0, 7, and 21 days after excision of femoral artery; **B** perfusion recovery in ND and KD group mice at 7, and 21 days after surgery; **C** representative original micrographs of hindlimb Sects. 14 days after surgery, CD31 ( +) stained are shown in green, DAPI-stained nuclei are shown in blue; **D** quantitative analysis of capillary density (Ratio of CD31 ( +) cells and DAPI ( +) cells; **E** relative gene expression of CD31 (PECAM) and VEGFA in ischemic hind limb 7 days after surgery; **F** protein expression of CD31 (PECAM) and VEGFA both in ischemic and non-ischemic limb 7 days after surgery. Mean ± SEM, n = 10 to 13, *P < 0.05, **P < 0.01, ***P < 0.001

To examine the effect of KD on perfusion recovery ability in chronic ischemic injury, a mouse ischemic limb model was used in mice fed both the KD and ND. Perfusion was assayed by laser Doppler perfusion imaging on days 0, 7, and 21 following femoral artery ligation surgery. We observed that the KD reduced perfusion signals associated with more pronounced non-perfusion signals in ischemic limbs than in mice fed with ND on days 7 and 21 (Fig. 2A). Quantitatively, the ratio of perfusion between ischemic and non-ischemic limbs for each mouse was calculated and it was found that the perfusion ratio was 86.2% at the 3-week point in the ND group, whereas it was only 52.4% in the KD group. There were no significant differences between the two groups on day 0, indicating slow recovery of limb circulation caused by KD (Fig. 2B). To assess the angiogenic effect, capillary density was measured. Using immunofluorescence staining, capillary density was detected using anti-CD31 antibody in the hindlimb (Fig. 2C). The micrographs showed that ischemic limbs of KD-fed mice displayed reduced capillary density (less red CD31 ( +) signal), indicating reduced angiogenesis (Fig. 2D). This was confirmed by western blot (WB) and quantitative PCR (qPCR) results of CD31 and vascular endothelial growth factor A (VEGFA), two common indicators of tissue revascularization [22]. By performing qPCR, mRNA level of both CD31 (platelet endothelial cell adhesion molecule-1) and VEGFA was found to be significantly reduced in the ischemic hind limb tissue of mice in the KD group compared with those in the ND group (Fig. 2E). Further WB tests showed that both CD31 and VEGFA protein expression levels significantly decreased in the hind limb tissue of KD mice following ischemic surgery compared with mice in the ND group, but no significant difference was found in limb tissue between the KD and ND groups before ischemic surgery, indicating that KD reduced revascularization in hind limb tissue after ischemia (Fig. 2F). Overall, these data indicate that KD impeded revascularization and blood perfusion of hind limb tissue after ischemia.

### KD induced muscle atrophy

**Fig. 3:**
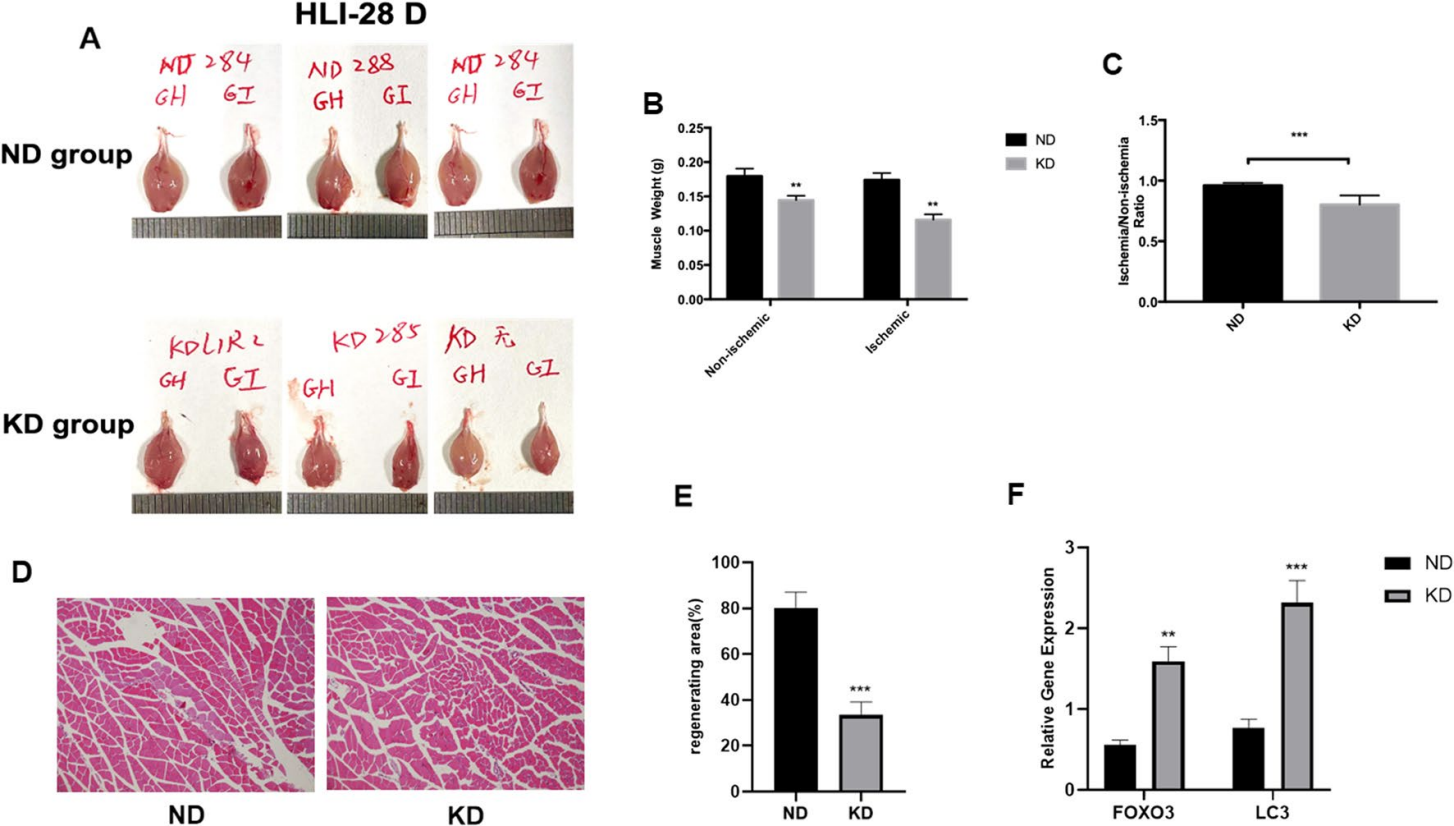
Quantitative analysis of gastrocnemius muscle atrophy. **A** images of ischemic limb and lateral limb muscle taken 28 days after surgery; **B** comparison of ischemic limb and lateral limb (non-ischemic) muscle weight between ND and KD groups, respectively; **C** comparison of ischemic limb/lateral limb muscle weight ratio between ND and KD groups; **D** representative hematoxylin–eosin staining images 28 days after hind limb surgery; **E** quantification of regenerating area, scale bar 10 mm; **F** relative gene expression of muscle atrophy related genes. Mean ± SEM, n = 10 to 13, *P < 0.05, **P < 0.01, ***P < 0.001

To assess the effect of KD on muscle regeneration and recovery following hind limb ischemia, we observed gastrocnemius muscle shape and weighed gastrocnemius muscle mass of both legs of mice fed KD and ND 28 days after surgery. An obvious muscle atrophy of gastrocnemius was observed on both legs of KD mice compared with those of mice in the ND group (Fig. 3A). To further confirm muscle atrophy in KD mice, gastrocnemius muscle mass was then assessed. The net weight of both ischemic and lateral gastrocnemius muscles of mice in the KD group was found to be significantly decreased compared with that of mice in the ND group, indicating that KD not only induced muscle atrophy of the ischemic limb but also caused atrophy in non-ischemic muscle (Fig. 3B). Muscle recovery rate was analyzed by calculating the ratio of ischemic gastrocnemius muscle weight to lateral gastrocnemius muscle weight of each mouse and the recovery ability of the gastrocnemius muscle after ischemia was found to be reduced in KD mice compared with mice in the ND group (Fig. 3C). Effect of KD on muscle regeneration ability was further assessed by H&E staining of the gastrocnemius muscle of the ischemic hind limb. There were irregular and small muscle fibers observed in ischemic hind limb tissue of mice in the KD group compared to mice in the ND group (Fig. 3D) while the calculated regenerating area based on H&E staining was found to be decreased in mice fed with KD (Fig. 3E). A previous study reported that KD induced muscle atrophy through muscle atrophy-related genes [33], therefore we further examined the expression level of muscle atrophy related genes FOXO3 and LC3 [23] in ischemic limb tissues. We found mRNA expression levels of both FOXO3 and LC3 genes were significantly increased in ischemic hind limb tissue of mice in the KD group (Fig. 3F) compared with mice in the ND group, indicating that upregulation of muscle atrophy related genes by KD may be one of the causes for muscle atrophy observed in our study.

### KD delayed wound healing and increased toe necrosis rate

**Fig. 4:**
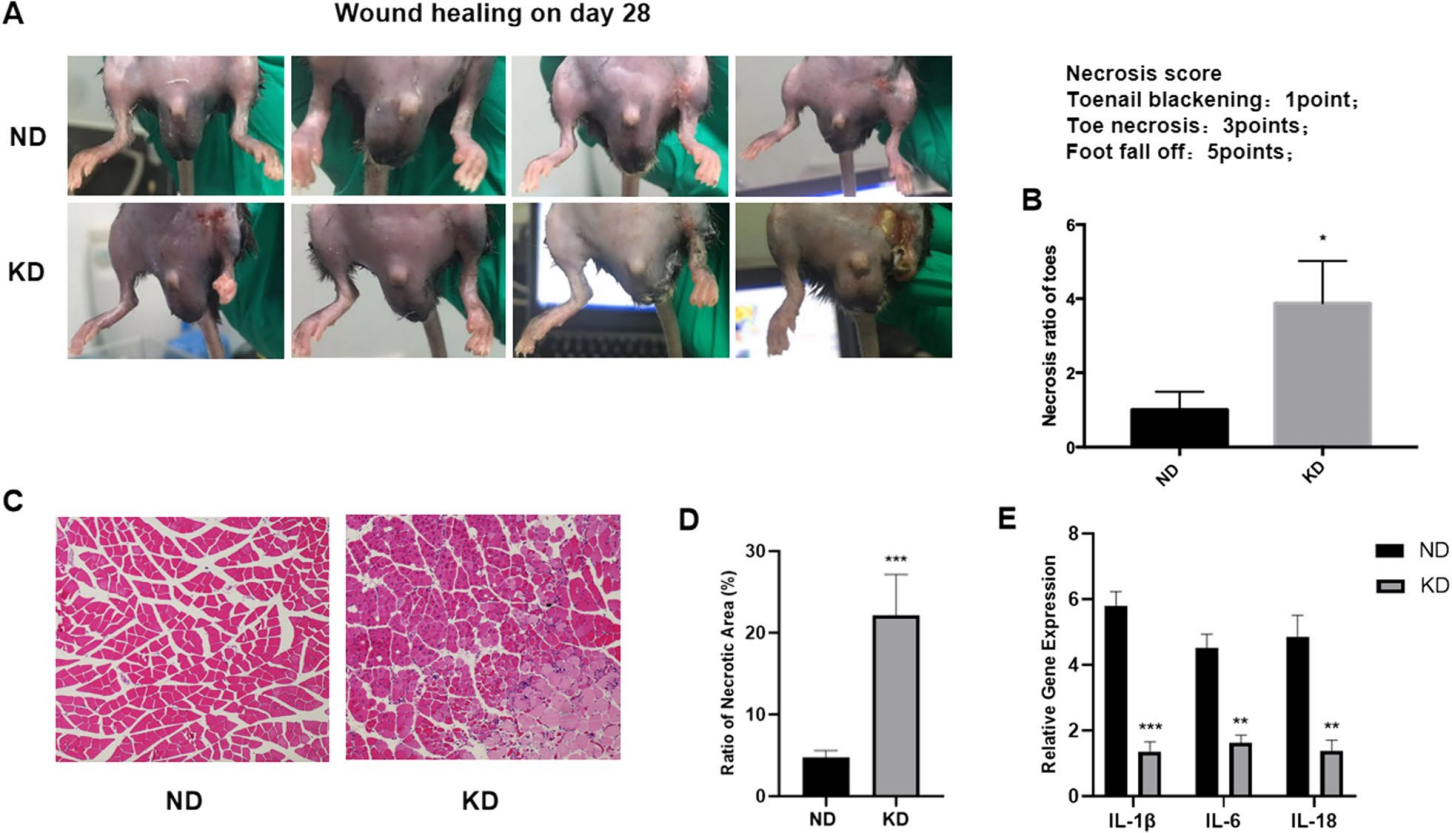
Effects of KD on wound healing in chronic hindlimb ischemia. **A** Image of surgical wounds 28 days after hind limb surgery; **B** comparison of necrosis ratio of toes between mice in the ND and KD groups; **C** representative hematoxylin–eosin (H&E) staining images 28 days after hind limb surgery; **D** comparison between ratio of necrotic area based on H&E staining in ND and KD group; **E** relative gene expression of inflammation related genes in ND and KD group respectively. Mean ± SEM, n = 8, *P < 0.05, **P < 0.01

We observed wound healing at the surgical site in each mouse and found that surgical wounds of mice in the ND group healed much faster than those of mice in the KD group. Images of wound closure in mice 28 days after hind limb surgery showed that the surgical wound of each mouse in the ND group healed completely when inflammatory and purulent exudation was observed at the surgical wound of mice in the KD group, indicating that KD significantly delayed wound healing and caused inflammation around the surgical sites (Fig. 4A). Severe toe necrosis in KD mice was also observed and the necrosis ratio of toes in the two groups was analyzed using necrosis score (1 point for toenail blackening, 3 points for toe necrosis, and 5 points for foot having fallen off). Ratio of toe necrosis of mice in the KD group was higher than mice in the ND group (Fig. 4B). H&E staining also revealed massive inflammatory cell infiltration in ischemic hind limb tissue in the KD group, while there was no sign of inflammation in the ND group (Fig. 4C). The ratio of necrotic area analysis based on H&E staining showed a significantly higher ratio of necrotic area in the KD group than in the ND group (Fig. 4D). According to a previous study, KD has anti-inflammatory effects by reducing the inflammasome (NLRP3) and inflammatory gene expression, such as IL-β and IL-6 [24]. Therefore, expression level of inflammation-related genes (IL-β, IL-6, and IL-18) was examined in the ischemic hind limb tissue of mice in the ND and KD groups and found that expression level of inflammatory genes was significantly decreased in ischemic limb tissues of mice in the KD group compared with mice in the ND group (Fig. 4E).

### KD induced ischemic limb tissue fibrosis

**Fig. 5:**
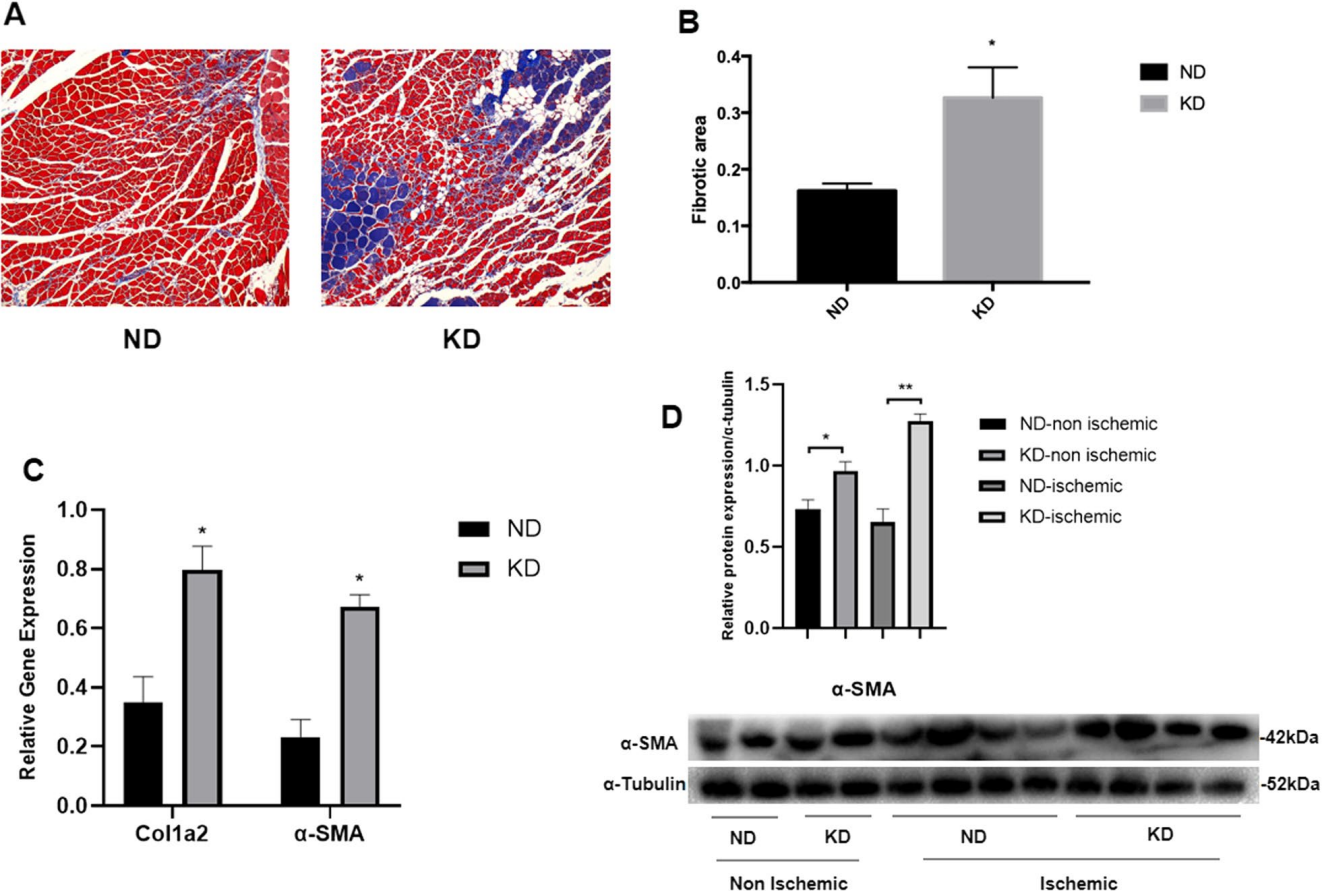
Effect of KD on fibrosis of hind limb tissue in chronic hindlimb ischemia. **A** Masson staining of ischemic limb tissue 28 days after hind limb surgery; **B** quantitative analysis of fibrotic area based on Masson staining; **C** relative gene expression of fibrosis related genes in ischemic tissues. **D** relative protein expression of α-SMA in both ischemic and non-ischemic tissues. Mean ± SEM, n = 8, *P < 0.05

Effect of KD on limb tissue fibrosis after ischemia was assessed by using Masson staining and examining fibrosis-related gene expression. Masson staining images showed severe fibrosis in the ischemic hind limb tissue of mice in the KD group compared to that of mice in the ND group (Fig. 5A). Additionally, analysis of the fibrotic area in the two groups based on Masson staining showed a significant increase in the fibrotic area of ischemic hind limb tissue of mice in the KD group compared with that of those in the ND group (Fig. 5B). Then gene expression level of Cola2 and α-SMA was measured to further evaluate fibrosis. Both Cola2 and α-SMA mRNA expression levels increased in the ischemic limb tissue of mice in the KD group compared with those in the ND group (Fig. 5C). Moreover, this was consistent with the finding of increased α-SMA protein expression in ischemic tissue of mice in the KD group (Fig. 5D). A slight increase in α-SMA protein expression was also found in non-ischemic tissue of mice in the KD group compared to that of mice in the ND group, indicating that KD can also trigger fibrosis without an ischemic condition.

### KD affected hind limb tissue metabolism both before and after ischemia at the genetic level

**Fig. 6:**
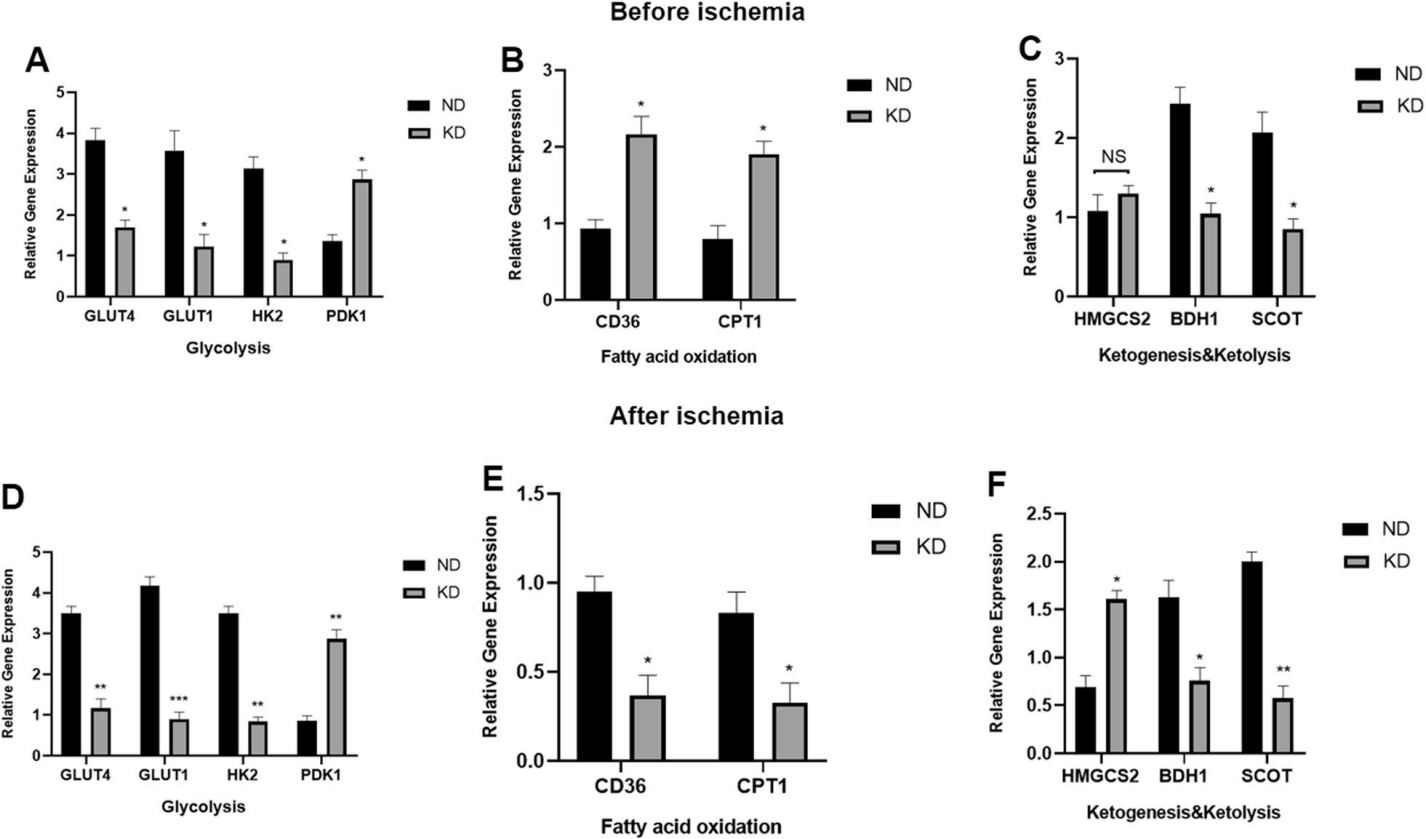
Metabolic changes in hind limb tissues at gene level before and after hind limb surgery. **A** Relative gene expression of glycolysis related genes in limb tissue before hind limb surgery; **B** relative gene expression of fatty acid oxidation related genes in limb tissue before hind limb surgery; **C** relative gene expression of ketone metabolism related genes in limb tissue before hind limb surgery; **D** relative gene expression of glycolysis related genes in limb tissue after hind limb surgery; **E** relative gene expression of fatty oxidation related genes in limb tissue after hind limb surgery; **F** relative gene expression of ketone metabolism related genes in limb tissue after hind limb surgery. Mean ± SEM, n = 8, *P < 0.05, **P < 0.01, ***P < 0.001

To understand the cellular impact of the KD, the metabolic status of hind limb tissues was investigated by examining metabolism-related genes, including those present during glycolysis (represented by GLUT4, GLUT1, HK2, and PDK1), fatty acid oxidation (represented by CD36 and CPT1), and ketone body metabolism (represented by HMGCS2, BDH1, and SCOT), before and after ischemic surgery. qPCR analysis of hind limb tissues before ischemia in the two groups of mice showed that KD significantly decreased glycolysis by decreasing GLUT4, GLUT1, and HK2 gene expression and increasing PDK1 gene expression (Fig. 6A), while it increased fatty acid utilization by increasing CD36 and CPT1 gene expression compared with ND mice (Fig. 6B). Ketolysis was simultaneously reduced by decreased BHD1 and SCOT gene expression in KD mice, while there was no significant difference in ketogenesis between the two groups represented by HMGCS2 gene expression (Fig. 6C). qPCR performed subsequently in ischemic limb tissues of mice in the two groups on day 7 after ischemic surgery showed that KD further decreased glycolysis in limb tissue after ischemia (Fig. 6D). In contrast to the result of increased CD36 and CPT1 gene expression found in non-ischemic tissue of mice fed with KD (Fig. 6E), they were decreased in limb tissue after ischemia indicating a decreased fatty oxidation by ischemia. The effect of KD on ketone metabolism in ischemic tissue was observed by further decreased BHD1 and SCOT gene expression indicating further decreased ketolysis and increased HMGCS2 gene expression indicating increased ketogenesis, which also differed from the results observed in non-ischemic tissue (Fig. 6F).

### KD affected hind limb tissue metabolism both before and after ischemia at the protein level

**Fig. 7:**
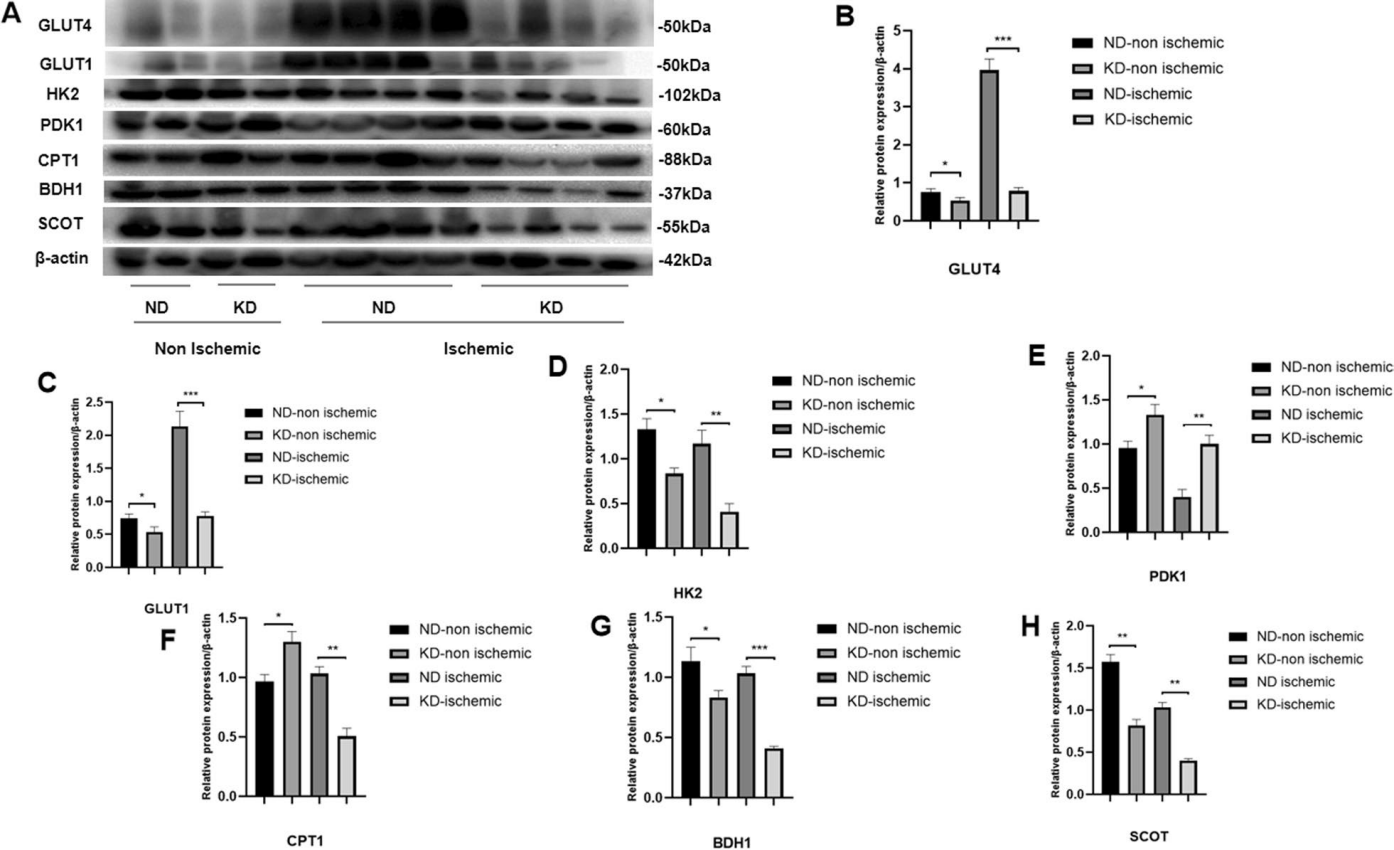
Metabolic change in hind limb tissues at protein level before and after hind limb surgery. **A** WB of hind limb tissue before and after hind limb surgery; **B** relative protein expression of GLUT4 in limb tissue before and after ischemic surgery and comparison between the two groups (ND and KD); **C** relative protein expression of GLUT1; **D** relative protein expression of HK2; **E** relative protein expression of PDK1; **F** relative protein expression of CPT1; **G** relative protein expression of BDH1; **H** relative protein expression of SCOT. Mean ± SEM, n = 8, *P < 0.05, **P < 0.01, ***P < 0.001

Metabolic changes caused by KD were further evaluated at the protein level in the hind limb tissues before and after ischemia. We performed WB (Fig. 7A) and found that KD decreased glucose uptake in the hind limb tissue of mice both before and after ischemia, as represented by GLUT4 and GLUT1, while a decrease was more significant in ischemic tissue (Fig. 7B, C). We further investigated how KD affects glycolysis, and found that expression of the HK2 protein, a glycolytic enzyme, was also decreased in the hind limb tissue of KD mice both before and after ischemia, but with a more significant decrease in ischemic tissue than that in mice in the ND group (Fig. 7D). However, PDK1 protein, an inhibitor of glycolysis, showed increased expression in hind limb tissue both before and after ischemia in KD mice, with a greater increase in ischemic tissue than that in mice in the ND group (Fig. 7E). The above results indicated that KD decreased glycolysis in hind limb tissue at the protein level, both before and after ischemia when it produced a greater decrease after ischemia. CPT1 protein expression was then examined, representing fatty acid uptake, and found to be increased in hind limb tissue of KD mice before ischemia, but decreased after ischemia compared with ND mice, indicating that KD increased fatty acid utilization of limb tissue before ischemia, and decreased its utilization under ischemic conditions (Fig. 7F). Thereafter expression level of BDH1 and SCOT proteins was examined, representing ketolysis of limb tissue. Both BDH1 and SCOT protein expression levels were decreased in limb tissue of mice both before and after surgery in the KD group, while the decrease was more significant in limb tissue after ischemia compared to that of mice in the ND group (Fig. 7G, H).

## Discussion

We conducted this experiment to observe how KD affects blood perfusion and tissue recovery after hind limb ischemia in mice. We found that KD impaired angiogenesis and blood recovery of hind limb tissue after ischemia in mice, induced muscle atrophy, and delayed wound healing. An aggravated inflammation and accelerated fibrosis was also observed in ischemic limb tissue in mice fed with KD. These findings indicate that KD impairs the blood recovery process of tissues under ischemia and increases the risk of delayed tissue recovery after an ischemia. It also highlights the possibility that muscle atrophy develops under a KD. Our results hint that patients with limb ischemia may need to avoid KD.

We found a significant decrease in the body weight of mice after 2 weeks of KD feeding. Effect of KD on weight loss was well established in previous studies and used as a tool to fight obesity in clinic at least in the short to medium term [25, 26]. But due to its poor maintainability, few clinical studies show long term interventions with a ketogenic diet, thus the further research is warranted. Previously Kozue et al. and Evan et al. reported that prolonged KD did not affect body weight in mice [27, 28], indicating that KD causes a rapid drop in body weight, but then it gradually returns to the initial level, and may even increase afterwards. There are several hypothesized mechanisms for its weight loss effect including reduction in appetite, reduction in lipogenesis and increased lipolysis, increased metabolic costs of gluconeogenesis and greater metabolic efficiency in consuming fats [29, 30], but how KD really causes weight loss still remains unknown. While the effect of KD on blood ketone levels is already established, its effect on blood glucose regulation is known to treat diabetes [31]. We found that KD causes a rapid change in blood metabolite levels at the beginning of the study then returns slightly towards the initial level and remains stable thereafter. The same fluctuation pattern of blood ketone level and blood glucose level was noticed in clinical trials [32]. Interestingly, we found that ketone content in the liver of KD mice was not significantly different from that mice in the ND group; however, ketone levels of muscle and heart tissues in KD mice were higher than those in ND mice,indicating an increased ketone metabolism in heart and muscle tissue of KD mice as they are known to be the major consumers of ketones.

Most importantly, we found that KD impaired angiogenesis and blood recovery after hind limb ischemia in mice, consistent with the finding of decreased angiogenesis in tumors by a calorie-restricted diet [18, 19]. An indispensable contribution of ketone metabolism to lymph vessel formation in vivo reported in previous study indicates that ketones are not merely a metabolite [21] so whether metabolic state under a KD effects angiogenesis of ischemic tissues needs to be investigated. In our study, we found that KD not only reduced blood perfusion in ischemic hind limb tissue but also decreased CD31 + number and downregulated the expression level of CD31 and VEGFA proteins, further indicating the reduced angiogenesis in ischemic limb tissues. Based on the results of reduced CD31 and VEGFA expression in ischemic limb tissue of KD mice, we hypothesized that endothelial cells might play a major role in the process of reduced angiogenesis; however the mechanism of KD,s effect on angiogenesis needs further studies.

We observed muscle atrophy of both ischemic and non-ischemic limb muscles in mice under a KD based on visual inspection of muscle shape and mass along with microscopic observations. Our findings are consistent with the results of another study which also reported that KD induces muscle atrophy in mice [33]. This study also reported that KD induced muscle atrophy in mice through upregulating muscle atrophy related genes; therefore we further assessed the expression level of these genes in ischemic limb tissue and found mRNA expression levels of both FOXO3 and LC3 genes were significantly increased in ischemic hind limb tissue of mice in the KD group, indicating that upregulation of muscle atrophy related genes by KD may be one of the causes for muscle atrophy observed in our study. However, whether KD also causes muscle waste during weight loss and further contributes to the muscle atrophy was not detected in our study, therefore it still needs further studies to determine the causes for muscle atrophy induced in mice under KD.

Reduced wound healing caused by calorie-restricted diet has been reported previously [34]; therefore we evaluated the wound healing after hind limb ischemia and observed that KD delayed wound healing in mice when it also increased the toe necrosis rate and induced severe inflammation at wound sites. Our findings of impaired wound healing and toe necrosis in mice can be explained by the reduced angiogenesis caused by KD. Anti-inflammatory effect of KD was established by previous studies through reducing inflamasomes and pro-inflammatory cytokines [24, 35]. Similarly, gene expression of pro-inflammatory cytokines (IL-β, IL-6, and IL-18) were found to be decreased in ischemic limb tissues of mice in KD group in our study, further supporting the anti-inflammatory effect of KD. However we found an aggravated infection at the wound site and accelerated inflammation in the ischemic limb tissue underneath in KD mice. Due to studies, reducing pro-inflammatory state of tissues is considered to be treatment for diseases those were under a prolonged pro-inflammatory state like diabetes, atherosclerosis, autoimmune disease and obesity [36]. However maintaining and even inducing pro-inflammatory state of tissue to kill pathogens may be crucial for treating infections, not to mention depletion of pro-inflammatory cytokines may increase the lethality during infections [37–40]. Thus reduced pro-inflammatory cytokines by KD is considered to be the reason for the aggravated inflammation and infections found in KD mice in our study, when it can also explain the impaired wound healing and toe necrosis.

A previous study reported that KD induced cardiac fibrosis in mice [41]; therefore, we evaluated fibrosis of ischemic limb tissue by Masson staining, and found that KD accelerated fibrosis in limb tissue after ischemia. We then found an increased gene expression of Cola2 and α-SMA followed by an elevated α-SMA protein level, with a further indication of fibrosis induced by KD. It needs further studies to determine the effect of KD on fibrosis and its potential mechanism.

Finally, we studied the metabolic status before and after ischemia by examining the gene and protein expression of metabolic enzymes and transporters. We found that KD significantly decreased glucose uptake by down regulating glucose transporter (GLUT1 and GLUT4) expression both at gene and protein levels in hind limb tissues, and this was further aggregated by ischemia. Same result was found for HK2, while PDK1 was upregulated by KD both at gene and protein levels, indicating that KD also decreases glycolysis in limb tissue. We also examined CD36 and CPT1 expression, and found that KD increased fatty acid uptake in limb tissue before ischemia but decreased its uptake after ischemia, indicating that ischemia interferes with the effect of KD on fatty acid uptake by limb tissue. Decreased ketolysis in muscle tissue in KD mice has been reported [27], thus we evaluated the effect of KD on ketone metabolism in limb tissue before and after ischemia, and we found that KD decreased ketolysis but did not affect ketogenesis of limb tissue before ischemia, while it further decreased ketolysis and increased ketogenesis after ischemia of limb tissue, indicating that ischemia accelerates KD’s impact on ketone metabolism. In our study we found that KD increased fatty acid oxidation while it decreased glucose and ketone utilization by limb tissue, but after ischemia KD overall decreased the catabolic metabolism in limb tissue, indicating that hypoxia caused by ischemia accelerated KD,s impact on catabolic metabolism of limb tissue; moreover we assume that the reduced energy metabolism of limb tissue may further contribute to the impaired tissue recovery of hind limb under ischemia. However the underlying mechanism of altered metabolism by KD under normoxic and hypoxic environment needs further studies.

## Limitations

We found weight loss on mice with a short term KD, but we did not observe its long term effect on body weight of mice when the food intake or calorie intake of mice should also be calculated in order to investigate the causes of wight loss induced by KD. Only male mice were used in our study, and it still needs further studies on both sex to identify whether KD,s impact on mice is sex related. To investigate the metabolic alteration of limb tissue we used gene and protein expression of metabolic enzymes while their enzymatic activity are also of important and needs precise evaluation in future studies.

## Supplementary Information

**Additional file 1.** Diet ingredient composition and primer sequences for qPCR.

## Data Availability

The data sets generated during and/or analyzed during the current 7 study are available from the corresponding author on reasonable request.

## References

[1] Dhamija R, Eckert S, Wirrell E (2013). Ketogenic diet. Can J Neurol Sci.

[2] Wheless JW (2008). History of the ketogenic diet. Epilepsia.

[3] Cahill GF (2006). Fuel metabolism in starvation. Annu Rev Nutr.

[4] Johnson RH, Walton JL, Krebs HA, Williamson DH (1969). Post-exercise ketosis. Lancet.

[5] Koeslag JH, Noakes TD, Sloan AW (1980). Post-exercise ketosis. J Physiol.

[6] Bolla AM, Caretto A, Laurenzi A, Scavini M, Piemonti L (2019). Low-carb and ketogenic diets in type 1 and type 2 diabetes. Nutrients.

[7] Walsh JJ, Myette-Côté É, Neudorf H, Little JP (2020). Potential therapeutic effects of exogenous ketone supplementation for type 2 diabetes: a review. Curr Pharm Des.

[8] Shukla SK, Gebregiworgis T, Purohit V, et al. Metabolic reprogramming induced by ketone bodies diminishes pancreatic cancer cachexia. Cancer Metab. 2014 Sep; 2:18. 10.1186/2049-3002-2-18. Erratum in: Cancer Metab. 2014; 2:22. PMID: 25228990; PMCID: PMC4165433. 10.1186/2049-3002-2-18 PMC4165433 25228990

[9] Poff AM, Ari C, Arnold P, Seyfried TN, D'Agostino DP (2014). Ketone supplementation decreases tumor cell viability and prolongs survival of mice with metastatic cancer. Int J Cancer.

[10] De Feyter HM, Behar KL, Rao JU, et al. A ketogenic diet increases transport and oxidation of ketone bodies in RG2 and 9L gliomas without affecting tumor growth. Neuro Oncol. 2016;18:1079–87. 10.1093/neuonc/now088. Epub 2016 May 3. PMID: 27142056; PMCID: PMC4933488. 10.1093/neuonc/now088 PMC4933488 27142056

[11] Goedeke L, Bates J, Vatner DF (2018). Acetyl-CoA carboxylase inhibition reverses NAFLD and hepatic insulin resistance but promotes hypertriglyceridemia in rodents. Hepatology.

[12] Rahman M, Muhammad S, Khan MA (2014). The β-hydroxybutyrate receptor HCA2 activates a neuroprotective subset of macrophages. Nat Commun.

[13] Al-Zaid NS, Dashti HM, Mathew TC, Juggi JS (2007). Low carbohydrate ketogenic diet enhances cardiac tolerance to global ischaemia. Acta Cardiol.

[14] Fitchett D, Zinman B, Wanner C, et al. Heart failure outcomes with empagliflozin in patients with type 2 diabetes at high cardiovascular risk: results of the EMPA-REG OUTCOME® trial. Eur Heart J. 2016;37:1526–34. 10.1093/eurheartj/ehv728. Epub 2016 Jan 26. Erratum for: Eur Heart J. 2016 May 14;37(19):1535–7. PMID: 26819227; PMCID: PMC4872285. 10.1093/eurheartj/ehv728 PMC4872285 26819227

[15] Prattichizzo F, De Nigris V, Micheloni S, La Sala L, Ceriello A (2018). Increases in circulating levels of ketone bodies and cardiovascular protection with SGLT2 inhibitors: Is low-grade inflammation the neglected component?. Diabetes Obes Metab.

[16] Kim DH, Park MH, Ha S (2019). Anti-inflammatory action of β-hydroxybutyrate via modulation of PGC-1α and FoxO1, mimicking calorie restriction. Aging.

[17] Viggiano A, Meccariello R, Santoro A (2019). A calorie-restricted ketogenic diet reduces cerebral cortex vascularization in prepubertal Rats. Nutrients.

[18] Mukherjee P, El-Abbadi MM, Kasperzyk JL, Ranes MK, Seyfried TN (2002). Dietary restriction reduces angiogenesis and growth in an orthotopic mouse brain tumour model. Br J Cancer.

[19] Lin BQ, Zeng ZY, Yang SS, Zhuang CW (2013). Dietary restriction suppresses tumor growth, reduces angiogenesis, and improves tumor microenvironment in human non-small-cell lung cancer xenografts. Lung Cancer.

[20] Xin B, Liu CL, Yang H (2016). Prolonged fasting improves endothelial progenitor cell-mediated ischemic angiogenesis in mice. Cell Physiol Biochem.

[21] García-Caballero M, Zecchin A, Souffreau J (2019). Role and therapeutic potential of dietary ketone bodies in lymph vessel growth. Nat Metab.

[22] Menger MM, Nalbach L, Roma LP (2021). Erythropoietin exposure of isolated pancreatic islets accelerates their revascularization after transplantation. Acta Diabetol.

[23] Mammucari C, Milan G, Romanello V (2007). FoxO3 controls autophagy in skeletal muscle in vivo. Cell Metab.

[24] Youm YH, Nguyen KY, Grant RW (2015). The ketone metabolite β-hydroxybutyrate blocks NLRP3 inflammasome-mediated inflammatory disease. Nat Med.

[25] Bueno NB, de Melo IS, de Oliveira SL, da Rocha AT (2013). Very-low-carbohydrate ketogenic diet v. low-fat diet for long-term weight loss: a meta-analysis of randomised controlled trials. Br J Nutr.

[26] Cincione RI, Losavio F, Ciolli F, Valenzano A, Cibelli G, Messina G, Polito R (2021). Effects of mixed of a ketogenic diet in overweight and obese women with polycystic ovary syndrome. Int J Environ Res Public Health.

[27] Shimizu K, Saito H, Sumi K (2018). Short-term and long-term ketogenic diet therapy and the addition of exercise have differential impacts on metabolic gene expression in the mouse energy-consuming organs heart and skeletal muscle. Nutr Res.

[28] Lien EC, Westermark AM, Zhang Y (2021). Low glycaemic diets alter lipid metabolism to influence tumour growth. Nature.

[29] Gibson AA, Seimon RV, Lee CM, Ayre J, Franklin J, Markovic TP, Caterson ID, Sainsbury A (2015). Do ketogenic diets really suppress appetite. A systematic review and meta-analysis. Obes Rev.

[30] Paoli A, Grimaldi K, Bianco A, Lodi A, Cenci L, Parmagnani A (2012). Medium term effects of a ketogenic diet and a mediterranean diet on resting energy expenditure and respiratory ratio. BMC Proc.

[31] Yancy WS, Mitchell NS, Westman EC (2019). Ketogenic diet for obesity and diabetes. JAMA Intern Med.

[32] Moreno B, Crujeiras AB, Bellido D, Sajoux I, Casanueva FF (2016). Obesity treatment by very low-calorie-ketogenic diet at two years: reduction in visceral fat and on the burden of disease. Endocrine.

[33] Nakao R, Abe T, Yamamoto S, Oishi K (2019). Ketogenic diet induces skeletal muscle atrophy via reducing muscle protein synthesis and possibly activating proteolysis in mice. Sci Rep.

[34] Hunt ND, Li GD, Zhu M, et al. Effect of calorie restriction and refeeding on skin wound healing in the rat. Age (Dordr). 2012;34:1453–8. 10.1007/s11357-011-9321-6. Epub 2011 Oct 27. Erratum in: Age (Dordr). 2012 Dec;34(6):1563. Miller, Marshall [added]. PMID: 22037865; PMCID: PMC3528375. 10.1007/s11357-011-9321-6 PMC3528375 22037865

[35] Deng Y, Xie M, Li Q, Xu X, Ou W, Zhang Y, Xiao H, Yu H, Zheng Y, Liang Y, Jiang C, Chen G, Du D, Zheng W, Wang S, Gong M, Chen Y, Tian R, Li T. Targeting Mitochondria-Inflammation Circuit by β-Hydroxybutyrate Mitigates HFpEF. Circ Res. 2021 Jan 22;128(2):232–245. 10.1161/CIRCRESAHA.120.317933. Epub 2020 Nov 12. Erratum in: Circ Res. 2022 Apr;130(7):e24. PMID: 33176578. 10.1161/CIRCRESAHA.120.317933 33176578

[36] Shapouri-Moghaddam A, Mohammadian S, Vazini H, Taghadosi M, Esmaeili SA, Mardani F, Seifi B, Mohammadi A, Afshari JT, Sahebkar A (2018). Macrophage plasticity, polarization, and function in health and disease. J Cell Physiol.

[37] Benoit M, Desnues B, Mege JL (2008). Macrophage polarization in bacterial infections. J Immunol.

[38] Burdo TH, Walker J, Williams KC (2015). Macrophage polarization in AIDS: dynamic interface between anti-viral and anti-inflammatory macrophages during acute and chronic infection. J Clin Cell Immunol.

[39] Bedi B, McNair NN, Förster I, Mead JR (2015). IL-18 cytokine levels modulate innate immune responses and cryptosporidiosis in mice. J Eukaryot Microbiol.

[40] Yang ML, Wang CT, Yang SJ, Leu CH, Chen SH, Wu CL, Shiau AL (2017). IL-6 ameliorates acute lung injury in influenza virus infection. Sci Rep.

[41] Xu S, Tao H, Cao W (2021). Ketogenic diets inhibit mitochondrial biogenesis and induce cardiac fibrosis. Signal Transduct Target Ther.

