# Supplementary material for: Effects of the ketogenic diet in mice with hind limb ischemia
Source: Nutr Metab (Lond). 2022 Aug 29;19:59. doi: 10.1186/s12986-022-00695-z (PMC9422126; doi:10.1186/s12986-022-00695-z)
Supplement: Supplementary file 1 — Additional file 1. Diet ingredient composition and primer sequences for qPCR. [file 12986_2022_695_MOESM1_ESM.docx]

**Supplementary Material**

**Effects of the ketogenic diet on hind limb ischemia in mice**

Adilan Shalamu^1,2,3*^, Zhen Dong^2,3,4*^, Bowen Liu^1,2,3^, Lihong Pan^2,3,4^, Yun Cai^1,2,3^, Xiurui Ma^5^,

Kai Hu^1^, Aijun Sun^1,2,3,4#^, Junbo Ge^1,2,3,4#^

1. Department of Cardiology, Zhongshan Hospital, Fudan University, Shanghai Institute of Cardiovascular Diseases, Shanghai, China

2. Key Laboratory of Viral Heart Diseases, National Health Commission, Shanghai, China

3. Key Laboratory of Viral Heart Diseases, Chinese Academy of Medical Sciences, Shanghai, China

4. Institutes of Biomedical Sciences, Fudan University, Shanghai, China

5. Department of Cardiology, Shan Xi Cardvascular Hospital, Taiyuan, 030024, China

*Contributed equally to this work; Email:Adeeleh@163.com

#Corresponding author; Email: [jbge@zs-hoppital.sh.cn;](mailto:jbge@zs-hoppital.sh.cn;) angelasunsh@163.com;

**Table1.Ingredient Composition**

**Ketogenic Diet** **Control Diet**

| Ingredient | gm | gm |
| --- | --- | --- |
| Casein | 163.8 | 94.21 |
| L-Cystine | 2.46 | 1.41 |
| Corn Starch | 0.00 | 349.51 |
| Maltodextrin | 0.00 | 32.97 |
| Sucrose | 0.00 | 382.48 |
| Cellulose | 81.9 | 47.1 |
| Soybean Oil | 40.95 | 23.55 |
| Butter | 624.08 | 18.84 |
| Miner MIal Mix S10026B | 81.9 | 47.10 |
| VitaminMix mix,V10001C,10x Vits | 1.64 | 0.94 |
| Choline Bitartrate | 3.28 | 1.88 |
| FD&C Red Dye #40 | 0.025 | 0 |
| FD&C Yellow Dye #5 | 0.025 | 0.025 |
| FD&C Blue Dye #1 | 0 | 0.025 |
| Total | 1000 | 1000 |

**Table 2. Primers for qPCR**

| Gene | Forward | Reverse |
| --- | --- | --- |
| PECAM1 | ACGCTGGTGCTCTATGCAAG | TCAGTTGCTGCCCATTCATCA |
| VEGFA | GCACATAGAGAGAATGAGCTTCC | CTCCGCTCTGAACAAGGCT |
| FOXO3 | CTGGGGGAACCTGTCCTATG | TCATTCTGAACGCGCATGAAG |
| [Map1lc3a](https://www.ncbi.nlm.nih.gov/gene/66734) | GACCGCTGTAAGGAGGTGC | CTTGACCAACTCGCTCATGTTA |
| IL-1β | GCAACTGTTCCTGAACTCAACT | ATCTTTTGGGGTCCGTCAACT |
| IL-6 | TAGTCCTTCCTACCCCAATTTCC | TTGGTCCTTAGCCACTCCTTC |
| IL-18 | GTGAACCCCAGACCAGACTG | CCTGGAACACGTTTCTGAAAGA |
| Col1a2 | TCGTGCCTAGCAACATGCC | TTTGTCAGAATACTGAGCAGCAA |
| aSMA | CCCAGACATCAGGGAGTAATGG | TCTATCGGATACTTCAGCGTCA |
| GLUT4 | GTGACTGGAACACTGGTCCTA | CCAGCCACGTTGCATTGTAG |
| GLUT1 | CAGTTCGGCTATAACACTGGTG | GCCCCCGACAGAGAAGATG |
| HK2 | TGATCGCCTGCTTATTCACGG | AACCGCCTAGAAATCTCCAGA |
| PDK1 | GGACTTCGGGTCAGTGAATGC | TCCTGAGAAGATTGTCGGGGA |
| CD36 | ATGGGCTGTGATCGGAACTG | GTCTTCCCAATAAGCATGTCTCC |
| CPT1 | GCACACCAGGCAGTAGCTTT | CAGGAGTTGATTCCAGACAGGTA |
| HMGCS2 | GAAGAGAGCGATGCAGGAAAC | GTCCACATATTGGGCTGGAAA |
| BDH1 | ACAAGACACACGCTGTTGTTT | CTCTTCAAGCTGTCCAGTTCC |
| SCOT | CATAAGGGGTGTGTCTGCTACT | GCAAGGTTGCACCATTAGGAAT |
| Actb | GGCTGTATTCCCCTCCATCG | CCAGTTGGTAACAATGCCATGT |
